# *Onosma bracteatum* Wall Aqueous–Ethanolic Extract Suppresses Complete Freund’s Adjuvant-Induced Arthritis in Rats via Regulation of TNF-α, IL-6, and C-Reactive Protein

**DOI:** 10.3390/molecules29081830

**Published:** 2024-04-17

**Authors:** Farah Zafar, Ghazala Shaheen, Hafiz Muhammad Asif, Mohd Farhan, Ghazala Muteeb, Mohammad Aatif

**Affiliations:** 1 Department of Eastern Medicine, University College of Conventional Medicine, Faculty of Medicine & Allied Health Sciences, The Islamia University of Bahawalpur, Bahawalpur 63100, Pakistan; ghazala.shaheen@iub.edu.pk (G.S.); doctor.asif101@gmail.com (H.M.A.); 2 Department of Chemistry, College of Science, King Faisal University, Al Ahsa-31982, Saudi Arabia; mfarhan@kfu.edu.sa; 3 Department of Basic Sciences, Preparatory Year, King Faisal University, Al-Ahsa 31982, Saudi Arabia; 4 Department of Nursing, College of Applied Medical Sciences, King Faisal University, Al-Ahsa 31982, Saudi Arabia; graza@kfu.edu.sa; 5 Department of Public Health, College of Applied Medical Sciences, King Faisal University, Al-Ahsa 31982, Saudi Arabia

**Keywords:** arthritis, *Onosma bracteatum* wall, inflammatory mediators, CFA, qRT-PCR

## Abstract

*Onosma bracteatum* Wall (*O. bracteatum*) has been used traditionally for the management of arthritis; however, its therapeutic potential warrants further investigation. This study aimed to evaluate the anti-arthritic effects of the aqueous–ethanolic extract of *O. bracteatum* leaves (AeOB) in a rat model of complete Freund’s adjuvant (CFA)-induced arthritis. Rats were treated with AeOB (250, 500, and 750 mg/kg), indomethacin (10 mg/kg), or a vehicle control from days 8 to 28 post-CFA injection. Arthritic score, paw diameter, and body weight were monitored at regular intervals. X-ray radiographs and histopathological analysis were performed to assess arthritic severity. Inflammatory cytokines tumor necrosis factor-alpha (TNF-α), interleukin-6 (IL-6), and C-reactive protein (CRP) were quantified by qPCR and icromatography. Phytochemical analysis of AeOB revealed alkaloids, flavonoids, phenols, tannins, Saponins, and glycosides. AeOB also exhibited antioxidant potential with an IC_50_ of 73.22 µg/mL in a DPPH assay. AeOB and diclofenac exhibited anti-inflammatory and anti-arthritic activities. Rats treated with AeOB at 750 mg/kg and indomethacin showed significantly reduced arthritic symptoms and joint inflammation versus the CFA control. The AeOB treatment downregulated TNF-α and IL-6 and decreased CRP levels compared with arthritic rats. Radiography and histopathology also showed improved prognosis. These findings demonstrate the anti-arthritic potential of AeOB leaves.

## 1. Introduction

Rheumatic disorders are a broad category of more than 100 autoimmune and chronic degenerative illnesses that are accompanied by constant or chronic pain, inflammation, and physical impairment [1]. Numerous biological processes, such as cell activation, differentiation, proliferation, and inflammation, are influenced by cytokines [2]. Defective innate and adaptive immune responses combine with chronic inflammation to induce cytokines [3]. The acute phase’s increased production of proinflammatory cytokines such as IL-1, TNF-α, NF-kB, and interleukin-6 ultimately results in bone deformation [2]. Along with increasing levels of proinflammatory cytokines, elevated levels of oxidative stress are significant elements in the pathophysiology of rheumatoid arthritis (RA), which harms the joints. These elements promote the release of reactive oxygen species (ROS) in synovial fluid; boost the formation of inflammatory cells, particularly neutrophils and macrophages; and cause further damage to the tissue [4]. Antioxidants protect cells from oxidative stress by preventing the development of reactive oxygen species in elements like proteins, lipids, and deoxyribonucleic acid, which are connected to pathologies including RA, diabetes, cancer, and Alzheimer’s disease [5].

The rheumatic illness burden is enormous and rising rapidly, mostly due to population increase and aging [1]. Between 0.5 and 1 percent of people globally have RA. According to reports, Pakistan has a 58–148/1000 prevalence of rheumatic illnesses, with at least 15 million people with rheumatic disorders [6]. The UK has reported a prevalence of 0.81 percent, whereas India has it at 0.75 percent [7]. Probable causes include injury leading to degenerative joint pain and abnormal metabolism, which can lead to gout, immune system malfunction, and systemic lupus erythematosus [8,9].

Medicinal plants are favored as an alternative to modern medications for treating RA patients’ pain and reducing side effects [10]. The herb *Peganum harmala* has long been used to treat rheumatoid arthritis (RA) and other autoimmune diseases [11]. In an in vivo anti-arthritic study using CFA in rats, oral administration of *P. harmala* extract for 21 days reduced C-reactive protein and rheumatoid factor levels, liver enzymes (alanine transaminase, aspartate transaminase, and alkaline phosphatase) and restored the immune system, serum prostaglandin-E2, and TNF-α in polyarthritic rats [11]. *Zingiber officinale* has demonstrated anti-rheumatic and anti-inflammatory properties by preventing the formation of PGs and leukotrienes [12]. *Withania somnifera* is a strong anti-inflammatory plant that works by systematically reducing IL-4, TNF-α, IL-5, and IL-6 levels. By inhibiting the nuclear signaling system (NF-B), *Arctium lappa* has been demonstrated to have an outstanding effect in the treatment of rheumatoid illnesses, as well as chronic inflammatory conditions [10]. A well-known herbal remedy for rheumatism and inflammation is *Glycyrrhiza glabra* (Licorice), which targets the cyclooxygenase-2 enzyme, a key participant in the etiology of tumors and RA [13].

*O. bracteatum* Wall is a plant species belonging to the Boraginaceae family. It is commonly known as “gaozaban” or “kaner” and is native to the Himalayan region, including India, Pakistan, and Nepal. This plant is widely used in traditional medicine for its therapeutic properties and is known for its anti-inflammatory, antitussive, anti-asthmatic, anti-tumor, and hepato-protective activities. The plant contains various bioactive compounds such as pyrrolizidine alkaloids, flavonoids, and triterpenoids, which are responsible for its medicinal properties. *O. bracteatum* has been extensively studied for its pharmacological activities. Several studies have reported its anti-inflammatory effects, which have been attributed to the presence of pyrrolizidine alkaloids [14].

However, no scientific data are available on the in vivo anti-inflammatory or anti-arthritic activity of *O. bracteatum* Wall. Therefore, the current study aimed to investigate the anti-arthritic potential of *O. bracteatum* Wall leaves in a CFA-induced arthritic rat model.

## 2. Results

### 2.1. Percentage Yield

The percentage yield of an aqueous–ethanolic extract of *O. bracteatum* Wall leaves was 6%.

### 2.2. Phytochemical Analysis

*O. bracteatum* Wall has been found to contain several classes of phytochemicals like alkaloids, flavonoids, tannins, phenolic compounds, etc. (Table 1).

### 2.3. Gas Chromatography–Mass Spectrometry (GC-MS) Analysis

**Figure 1:**
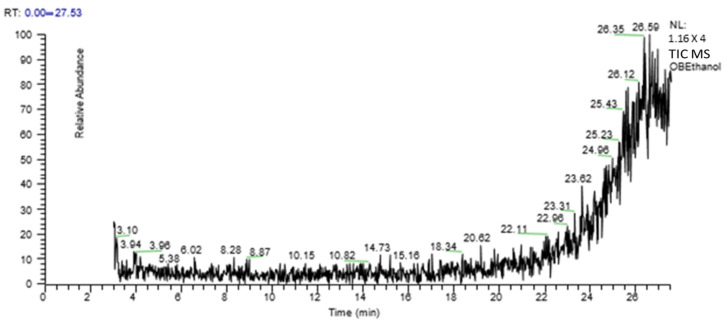
GC-MS chromatogram of aqueous–ethanolic extract of AeOB.

A GC-MS analysis of *O. bracteatum* Wall leaf aqueous–ethanolic extract showed eight compounds. In Table 2, the compound name, molecular weight, formula, and retention time (RT) are provided, and Figure 1 shows a chromatogram of AeOB. 

### 2.4. In Vitro Antioxidant Activity by 2,2-Diphenyl-1-Picrylhydrazyl Assay

**Figure 2:**
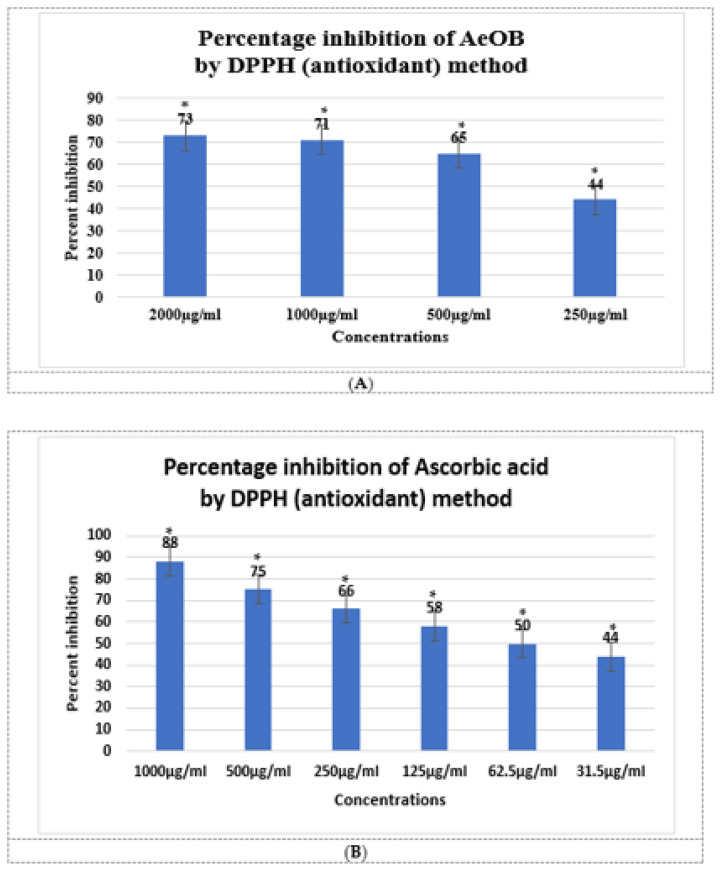
(**A**). Antioxidant potential of AeOB in DPPH assay. * shows *p* value is significant. Values shown are mean ± SD, n = 3. (**B**). Antioxidant potential of ascorbic acid in DPPH assay. Values shown are mean ± SD, n = 3.

**Figure 3:**
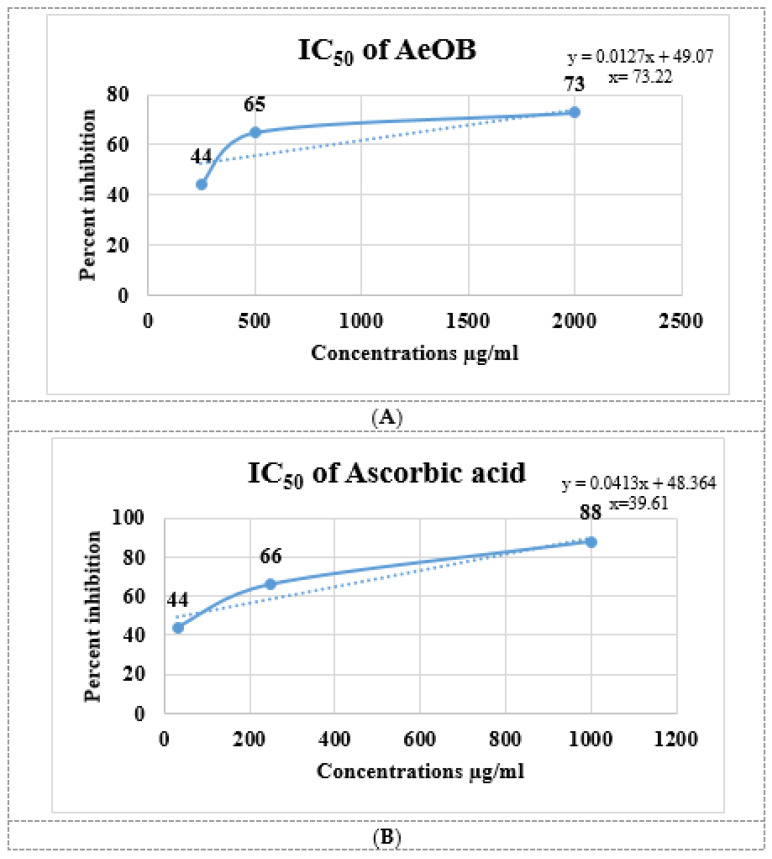
(**A**). IC_50_ of AeOB in DPPH assay. Values shown are mean ± SD in triplicate (n = 3). (**B**). IC_50_ of ascorbic acid (standard) in DPPH assay. Solid and dash lines are the trend line that provides a visual representation of the concentration-response relationship and are essential for determining the potency, efficacy, and mechanism of action of compounds in pharmacological studies.

The results of the antioxidant experiment showed that AeOB has relatively less free-radical-scavenging potentials than the ascorbic acid used as a standard, as shown in Figure 2A,B. The determined IC_50_ concentrations for AeOB and ascorbic acid were 73.22 and 39.61 µg/mL, respectively, as shown in Figure 3A,B.

### 2.5. In Vitro Anti-Inflammatory Activity

#### 2.5.1. Lipoxygenase (LOX) Inhibitory Assay

**Figure 4:**
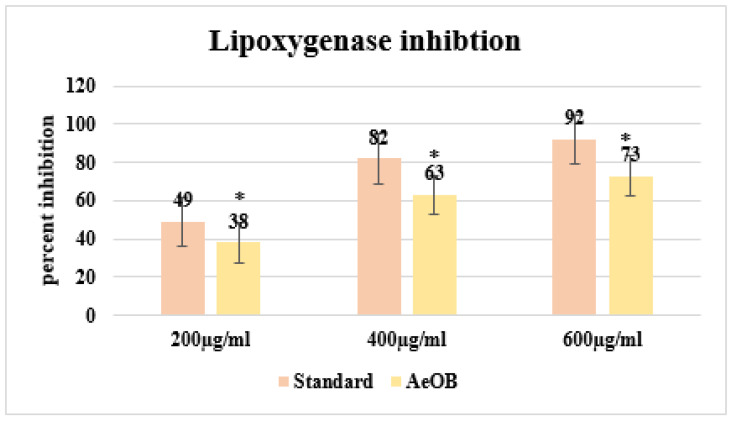
Percent inhibition by LOX inhibitory assay. * The mean difference is significant at the *p* ≤ 0.05 level. One-way ANOVA followed by LSD post hoc test was applied to check statistical significance.

After conducting the LOX inhibition assay, we were able to illustrate the shielding efficacy of AeOB and the standard (diclofenac sodium) through percentage values at concentrations of 200, 400, and 600 µg/mL. The details are provided in Figure 4. The comparison involves comparing these percentages with the standard drug Quercetin (with a *p*-value < 0.05), showcasing inhibition percentages of 92% at the corresponding highest concentration.

#### 2.5.2. Human Red Blood Cell (HRBC) Membrane Stabilization Method

**Figure 5:**
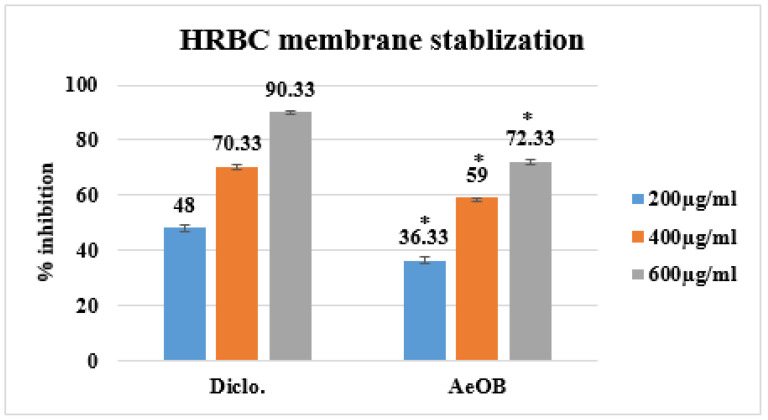
Percent inhibition by HRBC membrane stabilization method. * The mean difference is significant at the *p* ≤ 0.05 level. One-way ANOVA followed by LSD post hoc test was applied to check statistical significance.

Following the HRBC membrane stabilization method, the protective effects of AeOB and diclofenac sodium (standard) at concentrations of 200, 400, and 600 µg/mL are demonstrated as percentages in Figure 5. These percentages are in comparison with the standard drug diclofenac sodium (Diclo), which exhibits inhibition percentages of 90% at the highest corresponding concentration.

#### 2.5.3. Egg Albumin Denaturation Method

**Figure 6:**
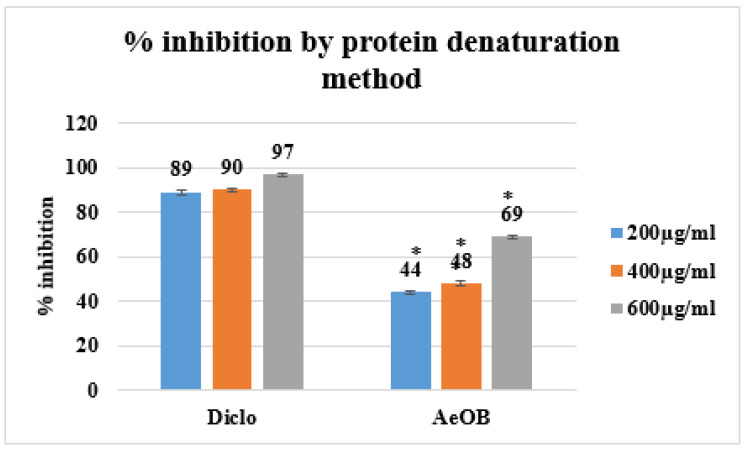
Percent inhibition by protein denaturation method. * The mean difference is significant at the *p* ≤ 0.05 level. One-way ANOVA followed by LSD post hoc test was applied to check statistical significance.

Following the protein denaturation method, the absorption percentages of AeOB and the standard at concentrations of 200, 400, and 600 µg/mL are demonstrated in Figure 6. These values are in comparison with the standard drug diclofenac sodium (standard), which exhibits an inhibition of 97% at the 600 µg/mL concentration.

### 2.6. Complete Freund’s Adjuvant-Induced Model Paw Diameter

**Figure 7:**
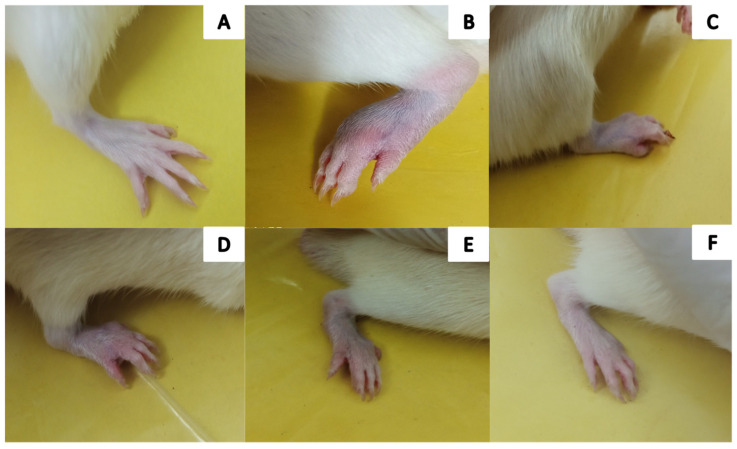
Visual illustration of CFA-induced rat paw on the 28th day: (**A**) Group-I (normal), (**B**) Group-II (arthritic control), (**C**) Group-III (standard), (**D**) Group-IV (AeOB, 250 mg/kg), (**E**) Group-V (AeOB, 500 mg/kg), and (**F**) Group-VI (AeOB, 750 mg/kg).

**Figure 8:**
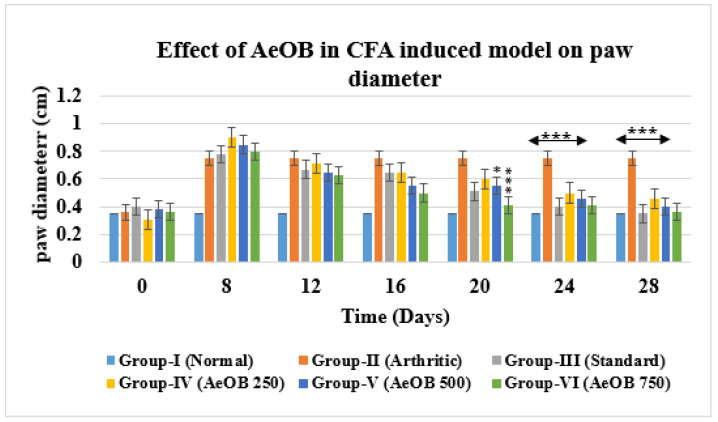
Reduction in paw diameter in different treatment groups compared with Group-II. Values are mean ± SD for n = 6. Stars indicate a comparison of Group-II with the treatment groups. At the * *p* ≤ 0.05 and *** *p* ≤ 0.001 levels, the mean difference is significant.

Severe CFA-induced arthritis was evident in the arthritic control group (Group-II) compared with Group-VI (AeOB, 750 mg/kg) and Group-III (indomethacin, 10 mg/kg). A reduction in the arthritic phase was observed from day 12 to day 28 in all groups except Group-II, as depicted in Figure 7. A reduction in paw diameter was also noted and was largest in Group-II compared with other treatment groups. However, the Group-VI (AeOB, 750 mg/kg) result was comparable to that of Group-III, as shown in Figure 8.

#### 2.6.1. The Visual Arthritic Scoring System

**Figure 9:**
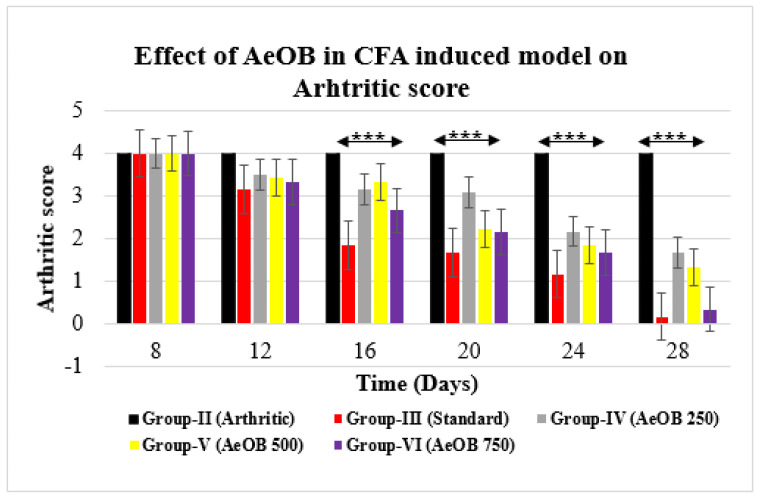
Reduction in arthritic score of different treatment groups compared with Group-II. Values are mean ± SD for n = 6. Stars indicate a comparison of Group-III and Group-VI with Group-II, respectively. The *** shows *p* ≤ 0.001, the mean difference is significant.

On the 28th day, the CFA model revealed that Group-II had a higher arthritic score. Group-VI- and Group-III-treated rats showed a substantial (*p* ≤ 0.001) decrease in the arthritic index on the 28th day compared with the arthritic control rats, as shown in Figure 9.

#### 2.6.2. Hot Plate/Thermal Hyperalgesia

**Figure 10:**
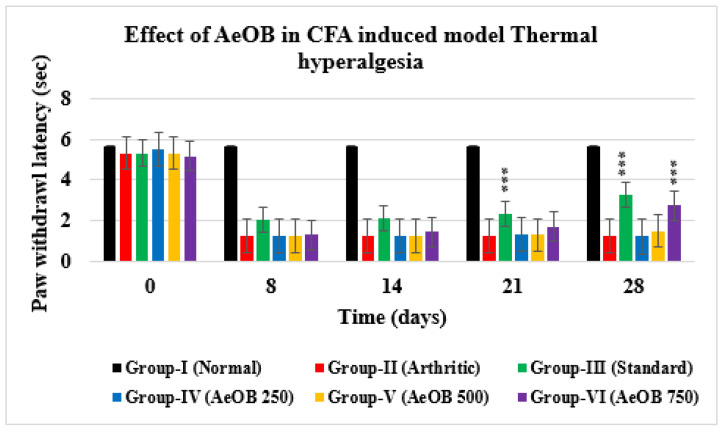
Paw withdrawal latency of different treatment groups compared with Group-II. Values are mean ± SD for n = 6. Stars indicate a comparison of Group-II with the other treatment groups. At the *** shows *p* ≤ 0.001 levels, the mean difference is significant.

Animals treated with 250, 500, and 750 mg/kg of AeOB and indomethacin showed a substantial increase in the paw withdrawal delay on days 0, 8, 14, 21, and 28, as shown in Figure 10. On the 28th day, it was found that the Group-VI paw withdrawal latency (2.75 ± 0.40) was comparable to that of Group-III (3.26 ± 0.70). However, it was found that Group-IV and Group-V paw withdrawal values (1.21 ± 0.27; 1.48 ± 0.14) were substantially shorter than Group-VI and Group-III values (*p* > 0.05).

#### 2.6.3. X-ray of Left Hind Paw

**Figure 11:**
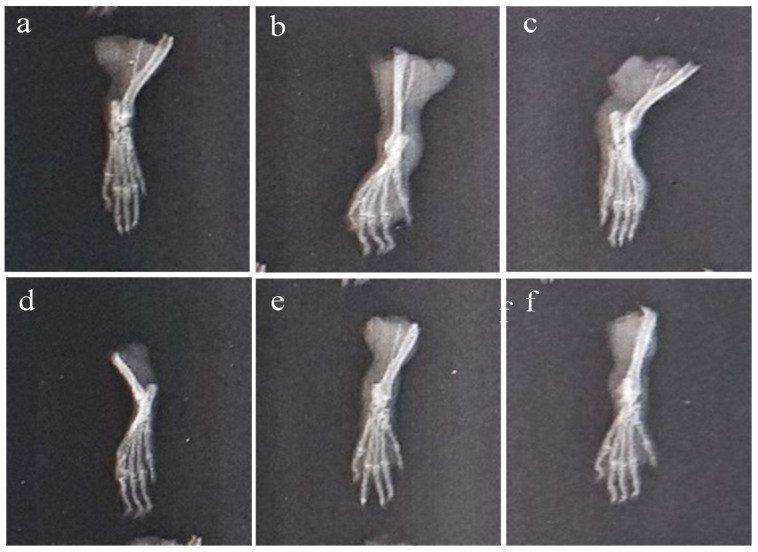
X-ray of CFA-induced rat paws: (**a**) Group-I (normal), (**b**) Group-II (arthritic control), (**c**) Group-III (standard), (**d**) Group-IV (AeOB, 250 mg/kg), (**e**) Group-V (AeOB, 500 mg/kg), and (**f**) Group-VI (AeOB 750 mg/kg) on day 28.

The paws’ X-ray radiographs on the 28th day are shown in Figure 11. This revealed extensive erosion, soft tissue swelling, and joint space narrowing (inter-tarsal joints) in Group-II. The use of plant extract and indomethacin, however, decreased the narrowing of joint space and enhanced the radiographic pattern of the joints. It was discovered that Group-VI animals had better radiographic patterns in the joints than the Group-IV animals.

#### 2.6.4. Body Weight

**Figure 12:**
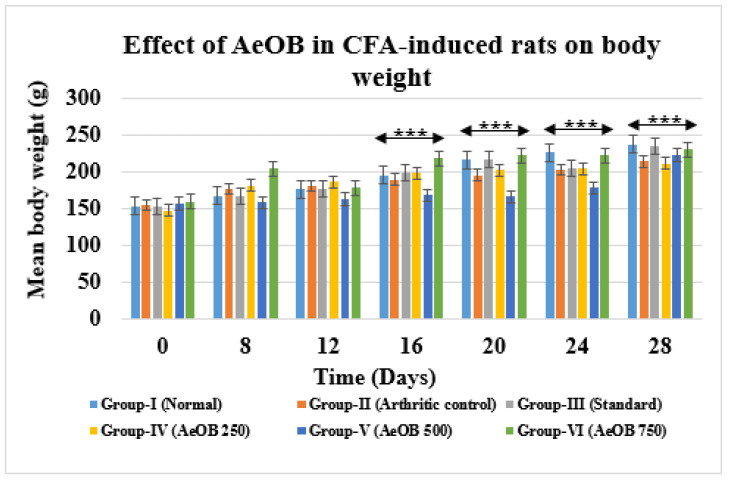
Values are mean ± SD and n = 6 for body weights in CFA-induced arthritic rats, *** = *p* ≤ 0.001. Stars indicate a comparison of the treatment groups with Group-I. At the *** *p* ≤ 0.001 level, the mean difference is significant.

From the 16th day onward, Group-III and Group-VI body weights increased as much as in Group-I (normal rats), which was significant at *p* ≤ 0.001 for Group-II, as shown in Figure 12. Overall, it was discovered that the rats’ body weights were positively impacted by both the standard and AeOB. 

#### 2.6.5. Effect of AEOB on Serum TNF-α, IL-6 Gene Expression, and CRP in CFA-Induced Arthritic Model

During the treatment, in Group-VI and Group-III, which received AeOB (750 mg/kg) and indomethacin (10 mg/kg), respectively, the serum fold change differences in TNF-α (0.36 ± 0.43; 0.004 ± 0.00) and IL-6 (0.002 ± 0.0; 0.001 ± 0.00) were lower compared with Group-II (1.0002 ±0.02, 1.0015 ± 0.06), which had considerably higher values, as shown in Table 3, indicating that the treatment plants downregulated IL-6 and TNF-α. The CRP value in Group-II was 4.92 ± 0.05 mg/dL, and it was discovered that this value was considerably lower in Group-VI (1.40 ± 0.03 mg/dL) and Group-III (1.09 ± 0.03 mg/dL), as shown in Table 4.

#### 2.6.6. Hematological and Biochemical Estimation

**Figure 13:**
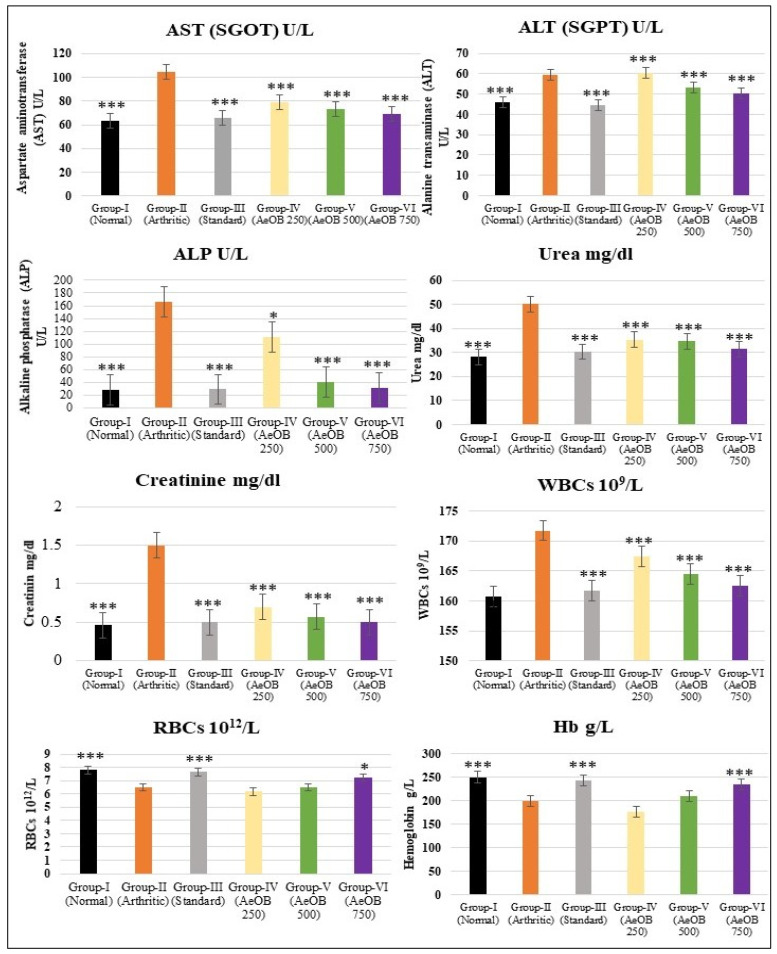
Values are mean ± SD and n = 6 for biochemical and hematological parameters in CFA-induced arthritic rats, where * = *p* ≤ 0.05, *** = *p* ≤ 0.001. Stars indicate a comparison of the arthritic control group with the other treatment groups.

The arthritic control group in the CFA-induced model displays signs of liver and kidney dysfunction, increased WBCs, and anemia. Treatment with indomethacin and AeOB, 750 mg/kg, appears to counteract these effects by reducing liver enzyme levels and WBCs and improving anemia parameters, as shown in Figure 13. The results are comparable to the standard drug indomethacin at *p* ≤ 0.001.

#### 2.6.7. Histopathology of Left Hind Paw

**Figure 14:**
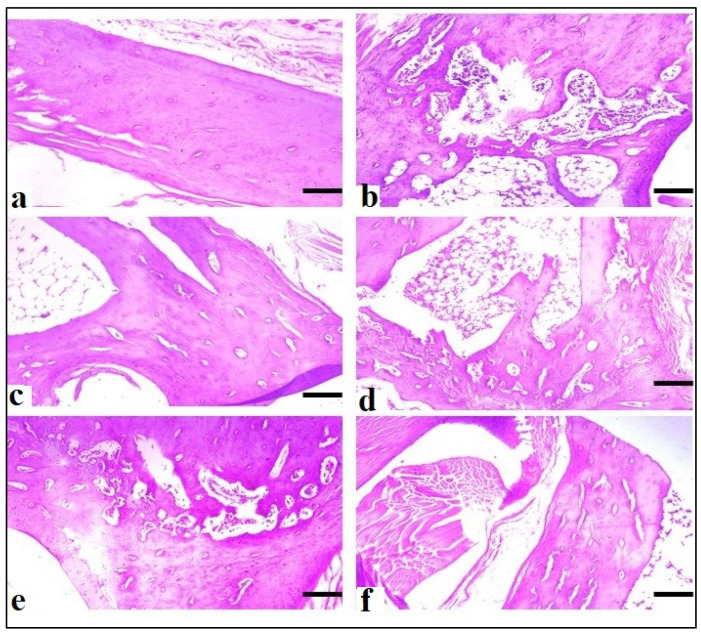
Microscopic evaluation at ×10 of CFA-induced rat paws: (**a**) Group-I (normal), (**b**) Group-II (arthritic control), (**c**) Group-III (standard), (**d**) Group-IV (AeOB, 250 mg/kg), (**e**) Group-V (AeOB, 500 mg/kg), and (**f**) Group-VI (AeOB, 750 mg/kg) on day 28 of treatment.

At the end of the CFA model on the 28th day, a histopathological analysis of the paws revealed that animals treated with AeOB (750 mg/kg) (Group-VI) and indomethacin (Group-III) had considerably fewer abnormalities compared with the arthritic control (Group-II), as shown in Table 5. Figure 14 shows microscopic images of the paws of different groups.

## 3. Discussion

The high incidence of arthritis in low- to middle-income countries makes it difficult for people to achieve their basic social and personal demands. Priority should be given to fundamental initiatives for arthritis management and prevention [24]. The majority of manufactured medications such as nonsteroidal anti-inflammatory drugs (NSAIDs), disease-modifying anti-rheumatic drugs (DMARDs), corticosteroids, and analgesics affect the symptoms but not the causes of the disease [25]. Surgical therapies can result in post-operative complications with extreme adverse pharmacological effects [26]. Physicians worldwide are interested in adopting natural products for the management of inflammatory disorders and pain due to the danger of the adverse effects of available treatments [27]. The current study examined the anti-inflammatory and anti-arthritic potential of AeOB in a CFA-induced arthritis rat model. The presence of phytochemicals with documented anti-inflammatory and antioxidant properties, such as matrine [16], Quercetin-4′-glucoside [21], Pyrrolidine [20], etc., was confirmed by a GC-MS analysis of AeOB. Plants have many valuable phytoconstituents that are essential to their biological actions. Flavonoids—in addition to having outstanding inhibition properties in prostaglandin-producing enzymes such as phospholipase A2, protein tyrosine kinase, and cyclooxygenases—are also powerful antioxidants [1] that can neutralize harmful free radicals in the body. By scavenging free radicals, flavonoids help protect cells from oxidative stress [28]. Additionally, phenols and glycosides may inhibit ROS, as well as inducible nitric oxide synthase (iNOS) pathways, from exerting their anti-inflammatory effects [10]. Membrane stabilization and protein denaturation are two widely employed methods used to evaluate the anti-inflammatory properties of compounds, particularly in the context of plant-derived substances. These methods provide valuable insights into the mechanisms through which bioactive compounds exert their anti-inflammatory effects. Previous studies have suggested that compounds exhibiting membrane-stabilizing properties may interfere with the release of phospholipases. Phospholipases play a crucial role in cellular responses, and their inhibition can mitigate the release of inflammatory mediators, thereby contributing to the overall anti-inflammatory activity of a substance [29]. In the current study, DPPH transformation from a violet to yellow color indicated that AeOB has the ability to scavenge free radicals, which are the cause of oxidative stress in RA. ROS can cause tissue damage and endothelial dysfunction by acting as signaling molecules in inflammatory diseases [30]. Therefore, it can be assumed that one of the primary mechanisms to suppress the expression of genes responsible for producing inflammatory cytokines and the cyclooxygenase enzyme (COX-2) in RA is a decline in oxidative stress caused by AeOB. Due to the CFA-induced arthritic model’s resemblance to human RA, it has been frequently used on rats for preclinical research [31,32]. The author Williams explores the utility of rodent models in understanding arthritis and their relevance to human disease. They discuss how rodent models help dissect disease mechanisms, test potential therapeutics, and identify biomarkers. Despite differences between rodent and human arthritis, these models remain valuable for studying disease pathogenesis and evaluating treatment strategies [32]. Similarly, JY Seo delves into the role of 7α,25-dihydroxycholesterol (7α,25-OHC) in osteoarthritis (OA) pathogenesis. They highlight how 7α,25-OHC triggers a cascade leading to chondrocyte death through oxiapoptophagy involving oxidative stress, apoptosis, and autophagy. The modulation of the p53-Akt-mTOR axis is implicated in this process, suggesting a potential therapeutic target for managing OA [33]. A study conducted by Ding et al. investigates the role of RUNX1 in rheumatoid arthritis (RA) progression and identifies its mechanism of action through the epigenetic inhibition of LRRC15. RUNX1 suppresses LRRC15 expression via epigenetic modifications, thereby mitigating RA progression. This finding suggests a potential therapeutic avenue for RA by targeting the RUNX1-LRRC15 axis [34]. CFA-induced arthritis has two phases: an acute phase that lasts 0 to 10 days and is caused by immune cells releasing histamine, serotonin, and prostaglandins and a chronic phase that lasts 11 to 28 days [35]. It has been noted that CFA stimulates the emission of IL-1, IL-6, and TNF-α from macrophages and monocytes. TNF-α also increases the release of IL-6 and IL-1, which leads to an escalation in leukocyte infiltration and vasodilation at the site of edema [36]. These pro-inflammatory cytokines also promote the production of chemokines, which draw neutrophils and monocytes to the damaged joints. The gene expression of matrix metalloproteinases is regulated by pro-inflammatory cytokines (TNF-α), which must be inhibited to prevent the degradation of bone and cartilage [27]. In the current study, after twenty-eight days of therapy, AeOB showed a statistically significant substantial decrease in paw edema, arthritic scores, and improvements in the histopathology of paws compared with the arthritic control group. According to previous studies, RA patients have higher levels of cytokines than healthy individuals, which are thought to be involved in cellular responses under inflammatory situations after being released by immune cells [37]. In our study, compared with the arthritic control, AeOB significantly reduced inflammatory cytokine IL-6 and TNF-α expressions. One of the clinical features of RA is anemia. Additionally, peri-articular osteoporosis and an increase in liver enzymes are linked to bone degeneration [27]. In our study, in rats with arthritis, AeOB was observed to restore the levels of liver enzymes. Additionally, AeOB kept hemoglobin levels stable in the treated groups. Recent studies have unveiled promising connections between the traditional medicinal herb *Onosma bracteatum* and its potential therapeutic effects on rheumatoid arthritis (RA). *Onosma bracteatum*, commonly used in traditional medicine systems like Ayurveda and Unani, contains bioactive compounds known for their anti-inflammatory and immunomodulatory properties [38]. This botanical remedy has shown notable efficacy in preclinical models of arthritis by attenuating inflammatory pathways and ameliorating joint destruction [39]. Furthermore, an elevated level of CRP is the main marker of systemic inflammation, indicating active inflammation. Increased CRP levels also signal a worse prognosis of arthritis. Previous studies have demonstrated that elevated IL-6 and TNF- levels exacerbate CRP production [40]. As a consequence, AeOB probably reduces CRP levels by reducing IL-6 and TNF-α, which shows that systemic inflammation is suppressed.

## 4. Material and Methods

### 4.1. Materials

*O. bracteatum* Wall plant leaves were purchased from the local market of Bahawalpur. Ascorbic acid, 2,2-diphenyl-1-picrylhydrazyl (DPPH), ethanol, sodium dihydrogen phosphate, and sodium hydroxide were purchased from Merck, Darmstadt, Germany. CFA was purchased from Zokeyo, Wuhan, China. Hydrochloric acid was purchased from Anala BDH Laboratory, London, UK. Deionized water was from an industrial research laboratory, the Islamia University of Bahawalpur, Bahawalpur, Pakistan. Normal saline, indomethacin from Nishtar Medical Store, was from Bahawalpur.

### 4.2. Experimental Animals

Male Albino Wistar rats (weighing 150–250 g) were procured from an animal house at the Islamia University of Bahawalpur. Animals were kept in a typical animal housing facility at 24 °C, a relative humidity of 45–50%, and a 12 h dark/light cycle. Rats were given a conventional pellet diet and water (ad libitum) [22]. Before beginning experimental investigations, all rats were allowed to adapt to the laboratory environment. The institutional animal ethics committee provided study protocol approval with study No.487/AS&R.

### 4.3. Plant Assortment

Leaves of *O. bracteatum* Wall were collected from the local market of Bahawalpur in September 2022 and were identified by Assistant Professor. Dr. Muhammad Sarwar, taxonomist at the Herbarium of Botany Department, Faculty of Life Sciences, the Islamia University of Bahawalpur, Pakistan. Voucher no. of *O. bracteatum* Wall was ref no.113.botany.

#### Method for Forming an Aqueous–Ethanolic Extract

The leaves of *Onosma bracteatum* were purchased in dried form (500 gm) from the Shdab Dawakhana local market, Bahawalpur, Pakistan. The collected plant was cleaned and washed with double distilled water and dried under shade. Coarsely ground plant material was passed through a no. 60 sieve. The net weight after processing was 400 gm, which was then soaked in 2 L (70%) ethanol (solute to solvent ratio 1:5) at 25 °C for six days. Stirring was performed on and off. Thereafter, the extract was filtered through muslin cloth followed by Whatman filter paper (grade-1). A rotary evaporator (Heidolph, TOKYO RIKAKIKAI Co., Ltd. (TN Koishikawa Bldg., 1-15-17 Koishikawa, Bunkyo-ku, Tokyo 112-0002, Japan)) was used to dry the filtrate at 40 °C and 30 rotations per minute to evaporate ethanol. The resultant extract was stored in an airtight glass jar in a dark and cool place (refrigerator) at 4 °C for further use and labeled as AeOB. The percentage was calculated by using Equation (1).

(1) $$\%yield=\frac{Weight\;of\;extract\;obtained}{Weight\;of\;raw\;plant}\times 100$$

### 4.4. Screening of Phytochemical

Standard procedures were used to determine various phytoconstituents like carbohydrates (Seliwanoff’s and Fehling tests), flavonoids (alkaline reagent test), alkaloids (Dragendroff and Wagner’s tests), glycosides (Keller–Killani test), tannins (bromine water and 10% NaOH tests), saponins (foam test), and phenols (iodine and FeCl_3_ tests) [41].

### 4.5. Estimation of Gas Chromatography–Mass Spectrometry (GC-MC)

Thermo Scientific (DSQII) GC was used to test AeOB. The GC was outfitted with a TR-5MS capillary column that measured 30 m in length, 0.25 µM in film thickness, and 0.25 mm in internal diameter. Helium (He) was the carrier gas, and the flow rate was 1 mL per mint. With a temperature of 250 °C, the injector was operated in split mode. A sample volume of 1 µL was injected with an initial oven temperature of 50 °C and held for 2 min, followed by temperature increases of 150 °C at a rate of 8 °C/min and 300 °C at a rate of 15 °C for another 5 min [42].

### 4.6. In Vitro Antioxidant Activity by 2,2-Diphenyl-1-Picrylhydrazyl Assay

DPPH was used to determine the antioxidant activity of AeOB using the previously established method by Ahmad et al. [43], with slight changes. Ascorbic acid was used as the standard. The total assay volume was 100 μL. DPPH 0.1 mM solution in methanol was prepared. In each well of a 96-well plate, 90 μL of DPPH solution, a tested sample of 10 μL (5 mg/mL in methanol), and different concentrations of AeOB were included to calculate the IC_50_ value. The reaction mixture was incubated for 30 min at 37 °C. The absorbance was taken at 517 nm with an ELISA microplate reader (Biotek Synergy HT) in triplicate. The following formula was used to calculate the % inhibition.

(2) $$Inhibition\%=\left(\frac{Abs\;of\;control-Abs\;of\;sample}{Abs\;of\;control}\right)\times 100$$

### 4.7. In Vitro Anti-Inflammatory Activity

#### 4.7.1. Lipoxygenase (LOX) Inhibitory Assay

With minor changes, a spectrophotometric assay for measuring LOX inhibition was implemented [44]. Based on the production of a combination of Fe^3+^/xylenol orange with absorbance at 560 nm, this assay assesses the inhibition of test samples’ lipoxygenase activity with linoleic acid. In Tris-HCl buffer, the substrate linoleic acid (final concentration, 140 µM) was produced (50 mM; pH 7.4). Except for the aqueous extracts, which were made directly as 2 mg/mL in the Tris-HCl buffer, other extract quantities were made in 100% DMSO and diluted to 2 mg/mL in the buffer. At 25 °C for 5 min, 40 microliters of the enzyme (LOX), diluted to a final concentration of 0.2 U/mL in ice-cold Tris-HCl buffer, was combined with 20 microliters of various test sample quantities (µg/mL) or Quercetin (a positive control). The mixtures were incubated at 25 °C for 20 min in the dark after linoleic acid (40 µL) was added. Adding 100 µL of freshly made FOX reagent [sulfuric acid (30 mM), xylenol orange (100 µM), and iron (II) sulfate (100 µM) in methanol/water] ended the test (9:1). The blank comprised the enzyme LOX and the buffer; a negative control was formed from LOX solution, Tris-HCl buffer, substrate, and FOX reagent. Substrate was added after the FOX reagent in the negative control. Absorbance was taken at 560 nm after 30 min of incubation at 25 °C. The lipoxygenase inhibitory potential was calculated with the following formula:

$$Inhibition\%=\left(\frac{Abs\;of\;control-Abs\;of\;sample}{Abs\;of\;control}\right)\times 100$$

#### 4.7.2. Human Red Blood Cell (HRBC) Membrane Stabilization Method

A blood sample from a healthy individual (informed consent was obtained from the subject) was taken to conduct an in vitro anti-inflammatory investigation. By adopting IFBDO (International Federation of Blood Donor Organizations) guidelines, the blood sample was collected and approved by the ethics committee at the Islamia University of Bahawalpur. The Declaration of Helsinki guidelines were also adopted for this study. After centrifugation at 3000× *g* rpm for 5 min, normal saline was used to wash the blood samples. The isotonic buffer solution was used to prepare a 10% *v*/*v* suspension (10 mM sodium phosphate buffer, pH 7.4) [45]. To make a reaction mixture of 2 mL total volume, 1 mL of red blood cell suspension (10%) was combined with 1 mL of the experimental samples at various concentrations. The reaction mixture was cooled to room temperature after incubation at 50 °C for 25 min. Centrifuging was performed again at 2500 rpm for 5 min; absorbance was measured at 560 nm using a spectrophotometer. The standard drug was diclofenac sodium. The control was a phosphate buffer solution.

The % inhibition was determined using the following equation:

% Inhibition = 100 × (A_C_ − A_S_)/A_C_

where A_C_ = control absorbance, and A_S_ = test sample absorbance.

#### 4.7.3. Egg Albumin Denaturation Method

An egg albumin denaturation assay was performed by adopting the procedure outlined by [45] with minor modifications. Regarding this, reaction mixtures were prepared by adding 2 mL of different concentrations of test samples, 0.2 mL of egg albumin, and 2.8 mL of phosphate buffer (pH = 6.5). At 37 °C, reaction mixtures were incubated for twenty minutes followed by heating at 70 °C for 5 min. Absorbance was taken at 660 nm with a spectrophotometer after cooling the reaction mixture to room temperature. The same quantity of egg albumin and phosphate buffer was used as the negative control with 2 mL of distilled water, diclofenac sodium was used as the standard, and % inhibition was calculated with the following formula:

% Inhibition = 100 × (A_C_ − A_S_)/A_C_

where A_C_ = absorption of the control sample, and A_S_ = absorption of the test sample.

#### 4.7.4. Complete Freund’s Adjuvant-Induced Arthritis Model

The CFA model established by Tiwari R et al. was adopted for in vivo anti-arthritic activity with slight modifications [24]. Albino Wistar rats of 150–250 g weight were divided into 6 groups, and 6 rats were placed in each group.

Group-I (normal control): Distilled water (vehicle) was provided orally (10 mL/kg) from day 8 to day 28.

Group-II (negative/arthritic control): In the left hind paw (sub-plantar surface), 0.1 mL of CFA was injected with the help of a 26-gauge needle.

Group-III (positive control): After induction with CFA, indomethacin was orally provided as a standard drug (10 mg/kg b.w) from day 8 to day 28.

Groups-IV-VI (AeOB groups): Different doses of AeOB (250, 500, and 750 mg/kg) were administered orally from day 8 to day 28.

#### 4.7.5. Paw Diameter

On the zeroth, eighth, twelfth, sixteenth, twentieth, twenty-fourth, and twenty-eighth days of the experiment, measurements of paw diameter using a Vernier caliper were performed.

#### 4.7.6. Visual Arthritic Scoring System

The severity of the arthritis was evaluated using the visual arthritis rating system. The arthritis score has a range of 0 to 4 using the following grading system:

No swelling = 0, mild swelling and erythema = 1, swelling and erythema = 2, severe swelling and erythema = 3, and gross deformity and inability = 4.

#### 4.7.7. Hot Plate/Thermal Hyperalgesia

The hot plate method was used to measure the thermal hyperalgesia and paw withdrawal latency of the injected paw shortly before the injection of CFA on the first day and afterward at several time intervals until the 28th day. The hot plate was retained at a temperature of 55 ± 5 °C when paws were put on it. The pain threshold’s endpoint was measured as the time it takes for the rat to lick its paws or leap in response to heat stimulation. To prevent tissue injury, a 20 s cutoff time was used.

#### 4.7.8. X-ray and Histopathology

The rats were anesthetized and sacrificed on day 28 of the experiment under ketamine anesthesia at 10 mg/kg. The left hind paws were preserved in 20% formalin for further X-rays of CFA-injected paws and histopathological analysis.

#### 4.7.9. Body Weight Measurements

Before CFA injection on the first day of the experiment and then at various intervals up to the 28th day, body weight was measured using a digital weighing scale. Body weight was taken for arthritic assessments.

% changes in weight were calculated with the following formula:

(3) $$\%\;weight\;change=\frac{W_{t}-W_{0}}{W_{t}}\times 100$$

where W_t_ is animal weight at various time intervals, and W_0_ is body weight at 0 days.

#### 4.7.10. Quantitative Real-Time Polymerase Chain Reaction (qRT-PCR)

The qRT-PCR procedure for IL-6 and TNF-α was performed according to the optimized method. qRT-PCR was executed in a SLAN-96P Real-Time PCR System (Sansure Biotech Inc., Ghangsha, China) with a 2X SYBR qPCR Mixture (Zokeyo, China) in a total reaction volume of 15 μL that included 10 μL of SYBR Green mix, primers at 0.5 μM each, and 1 μL of cDNA as the template. The relevant CTs of the samples were compared against disease control and control samples containing housekeeping genes (GAPDH). The amplification conditions were 95 °C temperature for 30 s and 40 cycles of 95 °C for 5 s and 60 °C for 20 s. Primers used in the procedure are listed in Table 6.

#### 4.7.11. Biochemical and Hematological Estimation

All animal groups’ retro-orbital punctures were performed on the 28th day, and many biochemical parameters, including urea, creatinine, alanine transaminase (ALT), alkaline phosphatase (ALP), and aspartate aminotransferase (AST) were determined using Micro Lab 300 with a DiaSYs kit. CBC and CRP were performed using a BIOBASE analyzer and an ichroma analyzer, respectively.

### 4.8. Statistical Analysis

Values were recorded as mean ± SD, n = 6. By using IBM SPSS statistics 20, a one-way analysis of variance (ANOVA) followed by an LSD post hoc test was applied to calculate the level of significance. Results were compared with the control group. Statistically, *p*-values ≤ 0.05, 0.01, and 0.001 were taken as significant.

## 5. Conclusions

The findings of this study investigating the effects of AeOB in a CFA-induced arthritis rat model show promising outcomes in alleviating inflammation associated with arthritis. The present study shows a substantial diminution in inflammatory cytokines IL-6 and TNF-α levels, as evidenced by quantitative qRT-PCR analysis. IL-6 and TNF-alpha are well-known markers of inflammation, and their reduction indicates a potential anti-inflammatory effect of AeOB. Demonstrating the ability of AeOB to suppress inflammatory cytokines and protect the liver highlights the potential of AeOB-based therapies in managing inflammatory conditions effectively. Further research and clinical trials are warranted to discover the underlying mechanisms, as well as to assess the long-term efficacy of AeOB as a potential treatment for arthritis and other inflammatory diseases. If successful, these findings may pave the way for more targeted and efficient therapeutic approaches, improving the quality of life for millions of individuals suffering from inflammatory conditions.

## Figures and Tables

**Table 1:** Qualitative phytochemical analysis of AeOB leaf aqueous–ethanolic extract.

Sr. NO.	Class of Secondary Metabolites	Result
1	Tannins	+
2	Flavonoids	+
3	Saponins	+
4	Alkaloids	+
5	Glycosides	+
6	Carbohydrates	+
7	Phenolic compounds	+

Note: “+” sign indicates a positive class of secondary metabolites present in AeOB.

**Table 2:** Identification of AeOB ethanolic extract compounds by GC-MS analysis.

S. No.	Compound	M.W	Formula	Class	RT	Area %	Biological Activities	Ref.
1.	Matrine	248	C_15_H_24_N_2_O	Alkaloid	3.94	3.09	Antitumor, anti-inflammatory	[15,16]
2.	3′,4′,5,7-Tetramethoxyflavone	342	C_19_H_18_ O_6_	Flavonoid	6.02	0.29	Antioxidant	[17]
3.	6-ethyl-2,3,5,7-tetrahydroxy, 1,4-Naphthoquinone	250	C_12_H_10_O_6_	Spinochrome	10.15	0.92	Anti-inflammatory, Antimicrobial	[18]
4.	Thebaine	311	C_19_H_21_NO_3_	Opiate alkaloid	10.82	0.64	Analgesic	[19]
5.	Pyrrolidine	207	C_14_H_25_N	Alkaloid	15.16	0.57	Anticancer, anti-inflammatory, antiviral, anti-tuberculosis	[20]
6.	Quercetin-4’-glucoside	464	C_21_H_20_O_12_	Flavonoid o-glycosides	15.65	0.54	Antioxidant, anti-inflammatory	[21]
7.	Cyclohexane, 1,1′-(2-methyl-1,3 propanediyl) bis-	222	C_16_H_30_	-	20.62	1.07	Antioxidant	[22]
8.	2,4,6-Cycloheptatrien-1-one,3,5-bis-trimethylsilyl-	250	C_13_H_22_OSi_2_	-	26.65	16.73	Antioxidant, antimicrobial	[23]

**Table 3:** Differences in fold changes in pro-inflammatory cytokines gene expressions in CFA induced arthritic rat model.

Time (Days)	Group-I (Normal Control)	Group-II (Negative)	Group-III (Standard)	Group-IV (AeOB, 250 mg/kg)	Group-V (AeOB, 500 mg/kg)	Group-VI (AeOB, 750 mg/kg)
TNF-α	0.5019 ± 0.03 *	1.0002 ± 0.02	0.004 ± 0.00 ***	7.32 07 ± 0.77 *	2.19 ± 0.39 ***	0.36 ± 0.43 ***
IL-6	0.0301± 0.00 ***	1.0015 ± 0.06	0.001 ± 0.00 ***	0.385 ± 0.04 ***	0.0052 ± 0.00 ***	0.002 ± 0.00 ***

Values are mean ± SD and n = 6 for pro-inflammatory cytokines genes in CFA-induced arthritic rats, where * = *p* ≤ 0.05, and *** = *p* ≤ 0.001. Black-colored stars indicate a comparison of the treatment groups with the arthritic control (Group-II). At the *** *p* ≤ 0.001 level, the mean difference is significant.

**Table 4:** Difference in CRP reduction in the arthritic group compared with treatment groups.

Time (Days)	Group-II (Normal)	Group-II (Negative)	Group-III (Standard)	Group-IV (AeOB, 250 mg/kg)	Group-V (AeOB, 500 mg/kg)	Group-VI (AeOB, 750 mg/kg)
CRP	1.00 ± 0.00	4.92 ± 0.05	1.09 ± 0.03 ***	3.84 ± 0.17 ***	2.50 ± 0.17 ***	1.40 ± 0.03 ***

Values are mean ± SD and n = 6 for CRP in CFA-induced arthritic rats, where *** = *p* ≤ 0.001. Black-colored stars indicate a comparison of the treatment groups with the arthritic control. At the *** *p* ≤ 0.001 level, the mean difference is significant.

**Table 5:** Severity of different histopathological changes in the cartilage of rat left hind paws after 28 days of treatment.

Histopathological Lesions	Groups					
	Group-I (Normal)	Group-II (Arthritic) Control	Group-III (Standard)	Group-IV (AeOB, 250 mg)	Group-V (AeOB, 500 mg)	Group-VI (AeOB, 750 mg)
Cartilage						
Cartilage matrix destruction	−	++++	+	+++	++	+
Degeneration and resorption	−	++++	+	+++	++	+
Inflammatory cell infiltration	−	++++	+	+++	++	+
Joint/synovial space narrowing	−	++++	+	+++	++	+
Cellular infiltration with granuloma	−	++++	+	+++	++	+
Edema	−	++++	+	+++	++	+
Macrophage infiltration	−	++++	+	+++	++	+
Synovial membrane						
Hyperplasia of covering cells	−	++++	+	+++	++	+

(−) (+) signs indicate (−) = absent/normal, + = mild, ++ = moderate, +++ = severe, and ++++ = very severe.

**Table 6:** List of primers used to estimate IL-6 and TNF-α through qRT-PCR.

Marker	Sequence	Forward/Reverse
TNF-α	5′-ATGGGCTCCCTCTCATCAGT-3′	Forward
	5′-GCTTGGTGGTTTGCTACGAC-3′	Reverse
IL-6	5′-CCCACCAGGAACGAAAGTCA-3′	Forward
	5′-ACTGGCTGGAAGTCTCTTGC-3′	Reverse

## Data Availability

The data that support the findings of this study are available from the corresponding authors upon reasonable request.
